# Long non-coding RNAs: Potential therapeutic targets for epilepsy

**DOI:** 10.3389/fnins.2022.986874

**Published:** 2022-10-06

**Authors:** Sen Liu, Min Fan, Meng-Die Ma, Jin-Fang Ge, Fei-Hu Chen

**Affiliations:** ^1^School of Pharmacy, Anhui Medical University, Hefei, China; ^2^The Key Laboratory of Anti-Inflammatory and Immune Medicine, Ministry of Education, Hefei, China; ^3^Anhui Provincial Laboratory of Inflammatory and Immunity Disease, Hefei, China

**Keywords:** lncRNAs, epilepsy, drug discovery, neuroinflammation, apoptosis, GABA

## Abstract

Epilepsy is a common and disastrous neurological disorder characterized by abnormal firing of neurons in the brain, affecting about 70 million people worldwide. Long non-coding RNAs (LncRNAs) are a class of RNAs longer than 200 nucleotides without the capacity of protein coding, but they participate in a wide variety of pathophysiological processes. Alternated abundance and diversity of LncRNAs have been found in epilepsy patients and animal or cell models, suggesting a potential role of LncRNAs in epileptogenesis. This review will introduce the structure and function of LncRNAs, summarize the role of LncRNAs in the pathogenesis of epilepsy, especially its linkage with neuroinflammation, apoptosis, and transmitter balance, which will throw light on the molecular mechanism of epileptogenesis, and accelerate the clinical implementation of LncRNAs as a potential therapeutic target for treatment of epilepsy.

## Introduction

Epilepsy (recurrent unprovoked seizures) is a chronic disorder of transient brain dysfunction caused by sudden, abnormal firing of neurons, characterized by unpredictably recurrent seizures involve part or the entire body, leading to severe developmental and functional effects (World Health Organization., 2022). There are about 70 million epilepsy patients worldwide, and infants and the elderly accounting for the largest number of cases (Thijs et al., 2019). Once diagnosed, the first-line treatments for epilepsy patients are antiepileptic drugs and anticonvulsants, which aim to control seizures *via* regulating neuronal excitability through balancing the abundance of transmitters, especially γ-aminobutyric acid (GABA) and glutamate (Glu). However, there are many adverse drug effects, and one-third of patients have drug-refractory epilepsy. Moreover, lack of professional medical care is still the leading cause of the increased risk of epilepsy (Singh and Trevick, 2016). About 130,000 people die of epilepsy each year, with an annual direct cost of 1736–5848 US dollars and indirect cost of 2037–8587 US dollars for each epilepsy patient (Allers et al., 2015; Singh and Sander, 2020). Thus, epilepsy has been a great public health issue worldwide, and there is a critical need for a greater understanding of the pathogenesis and therapeutic targets of epilepsy, based on which more effective and non-invasive therapies should be explored and developed.

Long non-coding RNAs (LncRNAs) are RNAs with a length greater than 200 nucleotides whose transcripts are not involved in coding proteins. Changes of LncRNAs can be measured by RNA sequencing technology. Up till now only a small amount of lncRNAs have been characterized thoroughly, however, their changes including overexpression, deficiency or mutation have been implicated in numerous human diseases (Esteller, 2011; Ang et al., 2020). Recently, the role of LncRNAs in epileptogenesis has been illustrated gradually, involving mainly with the pathophysiological process of neuroinflammation, neuronal apoptosis, and balance of transmitters. In this paper, we will provide a short but comprehensive description of the research progress about the role of LncRNAs in the pathogenesis of epilepsy.

## Overview of epilepsy

Epilepsy is one of the world’s oldest recognized conditions, the written records of which could be dated back to 4000 BCE (World Health Organization., 2022). In 2017, according to the onset characteristics, the international league against epilepsy (ILAE) classified epilepsy into four categories including focal, generalized, generalized combined with focal, and epilepsy of unknown classification (Scheffer et al., 2017).

Epilepsy is a multifactorial disease with a strong genetic predisposition, and comorbidities such as depression and cognitive impairments are increasingly taken as important etiological and prognostic markers of epilepsy (Manford, 2017). Evidence from clinical and experimental studies indicate that GABA is substantially involved in the mechanism and treatment of epilepsy, and the widely accepted “GABA-hypothesis” implies that a reduction of GABA-ergic inhibition would lead to the onset of epilepsy while an enhancement of GABA-ergic inhibition could result in an antiepileptic effect (De Deyn et al., 1990). Consistently, abnormal GABAergic function and complex changes in Glu signaling have been demonstrated in the brain of epilepsy patients and animal models, with a confirmed suppression effect of GABA agonists and an inducible effect of GABA antagonists on epilepsy (Pfisterer et al., 2020). Moreover, benzodiazepines and barbiturates, which are widely used antiepileptic drugs, exert their effect *via* enhancing GABA-mediated inhibition (Treiman, 2001). More recently, it has been reported that neuroinflammation and neuronal apoptosis are also the main causes of epilepsy (Henshall and Simon, 2005; Vezzani et al., 2019; Kumar et al., 2022).

Affecting ion channels or neurotransmitters, the antiseizure medication might suppress seizures in up to two-thirds of all individuals (Manford, 2017). However, apart from the poor tolerability, they could not alter the long-term prognosis, and long-term use of traditional antiepileptic drugs can induce many adverse effects, including central nervous system problems, idiosyncratic reactions, neurocognitive and psychiatric symptoms, and long-term complications (Alsfouk et al., 2020). On February 19, 2016 and June 25, 2018, the FDA approved two new drugs, Briviact and Epidiolex, for treatment of epilepsy, but there are still insurmountable side effects including liver damage, anorexia, and allergic reactions (U.S. Food and Drug Administration, 2022a,b). Thanks to the advances in brain imaging, it is more possible for doctors and clinical investigators to identify the structural and functional causes and consequences of the epilepsies (Duncan, 2022). Based on these, epilepsy surgery has been one of the most effective ways to achieve long-term seizure freedom in special individuals with drug-resistant focal epilepsy (Manford, 2017; Duncan, 2022), but it is not popularized widely due to the limitations of indications and techniques. However, with the improved understanding of the biological mechanisms of epileptogenesis including epigenetic determinants and pharmacogenomics, appears a great hope for better and precisive pharmacological and non-pharmacological strategies to forecast, modify, or even cure epilepsy (Manford, 2017; Proix et al., 2021; Duncan, 2022).

## Biological characteristics and functions of long non-coding RNAs

Long non-coding RNAs are transcribed by RNA polymerase II or III and have no structures of appreciable open reading frame (ORF) and Kozak consensus sequence. Besides the traits of diverse and numerous, LncRNAs are involved in numerous important biological phenomena, and their unique characteristics and functional roles have been reviewed detailed in other studies (Quinn and Chang, 2016; Gao et al., 2020; Nojima and Proudfoot, 2022). Similar with that of the messenger RNAs (mRNAs), a maturation process is needed for LncRNAs, and mature LncRNA molecules could be found in the nucleus, cytosol, and organelles including the mitochondria (Villa et al., 2019). Generally, LncRNAs in the nucleus are mainly involved in epigenetic and transcriptional regulation, while LncRNAs in the cytoplasm are often participated in the regulation of post-transcription, through which regulating the mRNA stability, protein translation, and the competitive endogenous RNA (ceRNA) network (Gao et al., 2020).

**FIGURE 1:**
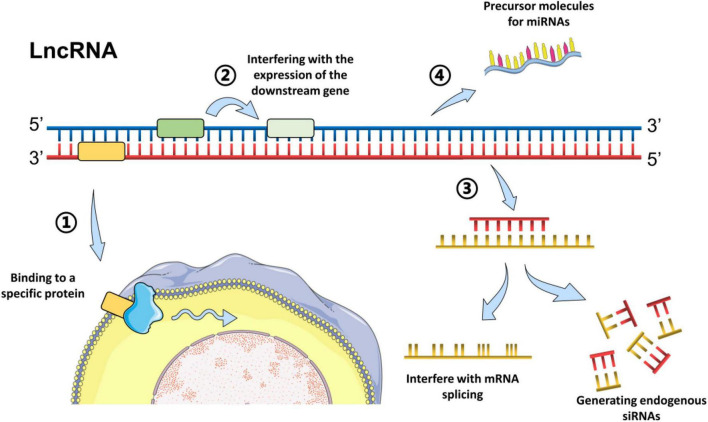
Common models of action of LncRNAs. Long non-coding RNAs can act through different biological mechanisms, mainly *via* methods as followings: **(1)** Bind to a specific protein and regulate its activity directly or indirectly by change the cellular localization *via* forming nucleic acid-protein complexes; **(2)** Encode the transcriptional process of the upstream promoter region of a protein gene, inhibit RNA polymerase II or mediate chromatin remodeling and histone modification, or interfere with the expression of the downstream gene. **(3)** Form complementary double strands with the transcription of the protein-encoding gene and generate endogenous siRNAs under the action of dicer enzyme, or interfere with mRNA splicing, and form different cleavage. **(4)** Serve as the precursor molecules for miRNAs (Grelet et al., 2017; Ali and Grote, 2020; Han et al., 2020; Zhang et al., 2020).

According to the position in the genome and their orientation to the adjacent protein-coding genes, LncRNAs can be subclassified into major groups including sense, antisense, intronic, intergenic, and bidirectional (Esteller, 2011). Generally, the LncRNA mechanisms of action can be divided into four categories: signal, decoy, guide, and scaffold, which has been described in detail in previous reviews (Gao et al., 2020; Nojima and Proudfoot, 2022). In brief, LncRNAs could regulate the transcription of downstream genes as signal molecules, or affect the expression of target genes by sponging miRNAs (Esteller, 2011). Moreover, LncRNAs could block a certain molecular pathway and act as a decoy molecule. Furthermore, LncRNAs can guide specific proteins to reach target location, or facilitate the interaction of numerous molecules and proteins, promoting the convergence and integration of information among different signaling pathways (Esteller, 2011; Quinn and Chang, 2016; Gao et al., 2020). For example, LncRNA H19 has been reported to inhibit glial cell activation in the hippocampus of epileptic rats by targeting STAT3 signaling way (Han et al., 2020), while LncRNA MEG3 could reduce neuronal apoptosis in the hippocampus of epileptic rats through the PI3K/AKT/mTOR pathway (Zhang et al., 2020). The common models of LncRNAs action are summarized in Figure 1.

It is worth to mention that apart from regulating the target gene expression at the transcriptional and post-transcriptional level, some LncRNAs could manipulate the expression of the target gene in an epigenetic way (Tsai et al., 2010; Peschansky and Wahlestedt, 2014). Serving as scaffolds, LncRNA could recruit chromatin remodeling complexes to a special combining site, or assemble select histone modification enzymes, thereby specifying the pattern of DNA methylation or histone status (Tsai et al., 2010; Chen and Xue, 2016). Through binding to the enhancer of zester homolog 2 (EZH2), which is a subunit of polycomb repressive complex 2 (PRC2), LncRNA GAS5 has been reported to guide EZH2 to the promoter of interferon response factor 4 (IRF4) and suppress its transcription, eventually leading to an inhibition of microglial M2 polarization (Sun et al., 2017). Acting as a modular scaffold of WDR5 and KAT2A complexes, LncRNA GClnc1 could promote gastric carcinogenesis *via* altering the pattern of histone modification (Sun et al., 2016). Moreover, it has also been reported that the HOX transcript antisense RNA (HOTAIR) could bind to the Polycomb Repressive Complex 2 (PRC2) and recruit it to target genes such as HOXD, then suppress the transcription of target gene *via* its histone methylase activity (Tsai et al., 2010). Otherwise, HOTAIR could bind to LSD1, which is a histone demethylase enzyme and part of the CoREST/REST repressor complex. *Via* the effect of LSD1, HOTAIR could remove the transcription-activating histone marks and reinforce the repressive activity of the PRC2 complex eventually (Tsai et al., 2010).

## Role of long non-coding RNAs in the progress of epilepsy and the possible mechanism

With the development of reliable technologies and useful tools, our understanding of LncRNAs implicated in epilepsy has made substantial progress. Involved with the fundamental cellular processes *via* regulating gene expression at epigenetics, transcription, and post-transcription, LncRNAs attribute substantially to the molecular mechanisms of epilepsy development. Results of a genome-wide LncRNA profiling study have demonstrated a differential regulation of LncRNAs in pilocarpine or kainate acid-induced temporal lobe epilepsy animal models (Lee et al., 2015; Jang et al., 2018). Using methylated DNA immunoprecipitation and hybridization technique, Miller-Delaney SF et al. detected and analyzed the methylation status of all annotated C-phosphate-G islands and promoter regions in the human genome, and established a hippocampal methylation profile of patients with temporal lobe epilepsy (Miller-Delaney et al., 2015). They found a total of 146 protein-coding genes exhibited altered DNA methylation in the hippocampus of temporal lobe epilepsy patients, with a close relation with the development processes, neuron maturation and remodeling (Miller-Delaney et al., 2015). Besides these findings, methylation-sensitive microRNAs were also identified in their study, including MiR27A, miR-193a-5p (MIR193A), and miR-876-3p (MIR876). More importantly, they reported the differential methylation of LncRNA in the hippocampus of epilepsy patients for the first time, including urothelial cancer associated 1 (UCA1), adenosine deaminase, RNA-specific, B2, antisense RNA 1 (ADARB2-AS1), long intergenic non-protein coding RNA 324 (LINC324) and mitogen-activated protein kinase 14, and antisense RNA 1 (MAP3K14-AS1) (Miller-Delaney et al., 2015).

Importantly, LncRNAs might be potential drug targets for treatment of epilepsy (Luo et al., 2019), which was supported by the results that administration of an antagoNAT (natural antisense transcript) could mediate an upregulation of the sodium channel protein SCN1A in a mice model of the epilepsy-related disorder, leading to significant improvements in seizure phenotype and excitability of hippocampal interneurons (Hsiao et al., 2016).

### Long non-coding RNAs and neuroinflammation in epilepsy

Neuroinflammation is a contributing factor for many neuropsychiatric diseases including Alzheimer’s disease and depression (Fang et al., 2020; Qi et al., 2021; Liu S. et al., 2022), and increasing evidence have demonstrated its role in the pathogenesis of epilepsy (Vezzani et al., 2019; Kumar et al., 2022). Using single-cell cellular indexing of transcriptomes and epitopes by sequencing (CITE-seq) techniques, Kumar et al. (2022) uncovered a pro-inflammatory microenvironment in the brain lesions of epileptic patients, including an extensive activation of microglia and an infiltration of other pro-inflammatory immune cells, together with a direct interaction between T cells and microglia. It is well known that activated microglia and astrocytes could interact with surrounding cells by secreting inflammatory factors, promoting neuroinflammatory responses into a vicious circle (Ginhoux et al., 2010; Tian et al., 2012; Santiago et al., 2017; Fang et al., 2020; Liu S. et al., 2022). Through microarray screening, Sun et al. (2017) identified that LncRNA GAS5 could regulate the polarization of microglia, acting as an epigenetic regulator. In a bilateral chronic constriction injury rat model, the expression levels of LncRNA 00311 and Lnc-AK141205 were upregulated in the microglia of dorsal spinal. Regulated by the STAT3 pathway, they were closely associated with the expression of IL-1β, IL-6, and TNF-α (Pang et al., 2020). Moreover, results of clinical investigation disclosed a relationship between cerebrospinal fluid IL1-β levels and an allelic variant of the IL1-β gene to the risk of developing epilepsy (Diamond et al., 2014). Furthermore, targeting at the inflammatory mediators in epilepsy, anti-IL-1, anti-IL-6, and anti-CD20 agents have been used in patients with drug-resistant epilepsy and refractory status epilepticus, with promising results and a good safety profile (Costagliola et al., 2022).

Through interacting with proteins, RNA, and DNA, LncRNA could serve as regulators of gene expression pathways involved with the regulation of both the pro-inflammatory and the anti-inflammatory process in the central nervous system (Tripathi et al., 2021). Acting as regulators of inflammation and neuronal differentiation pathways in the epileptic brain, LncRNAs are reported to be involved in the development of epilepsy (Zhao et al., 2019). Results of pre-clinical studies showed that the expressions of LncRNA H19 and LncRNA X inactive specific transcript (XIST) were up-regulated, while the expressions of LncRNA PVT1, LncRNA CACS2, and LncRNA UCA1 were down-regulated in epilepsy animal or cell models (Han et al., 2018a; Wang et al., 2020; Zhang et al., 2021). LncRNA H19 could promote an abnormal activation of rat hippocampal glial cells by targeting STAT3 signaling pathway *via* binding to miRNA-let-7b (Han et al., 2020), while LncRNA XIST has been reported to promote astrocyte activation through negatively regulating the expression of NEAT5 by binding to miR-29c-3p (Zhang et al., 2021). The down-regulated LncRNA PVT1, LncRNA CACS2, and LncRNA UCA1 could, respectively, regulate the downstream proteins including BDNF, PTEN, or JAK to promote the activation of astrocytes and the progression of epilepsy (Zhao et al., 2019; Wang et al., 2020; Zhu et al., 2020). Moreover, LncRNA UCA1 could inhibit the inflammation *via* regulating miR-203 mediated regulation of MEF2C/NF-κB signaling in epilepsy (Yu et al., 2020).

Long non-coding RNA growth arrest-specific 5 (GAS5) was markedly upregulated in epileptic cell and animal models, together with a down-regulated expression of miR-219. Moreover, GAS5 could epigenetically suppress the transcriptional miR-219 expression, and knockdown of GAS5 could dramatically promote the cell proliferation of epileptic cells and suppress the inflammation and apoptosis processes, which could be reversed by inhibition of miR-219, suggesting a regulation role of LncRNA GAS5 in the inflammatory response of epilepsy *via* its effect on miR-219 (Zhao et al., 2022). Cai et al. (2020) reported an elevated expression of LncRNA ILF3-AS1 in the serum and hippocampus tissue of epilepsy patients, which promoted the abnormal activation of astrocytes by specifically targeting at miR-212 and up-regulating the expression of inflammatory molecular such as tumor necrosis factor-α (TNF-α), IL-1β, and IL-6. Serum levels of LncRNA ZFAS1 were also reported higher in temporal lobe epilepsy patients, and ZFAS1 could exacerbate epilepsy development *via* promoting inflammation (He et al., 2021). Moreover, it has been demonstrated that transfection of pcDNA3.1- ZFAS1 could boost NF-κB activation and elevate the release of cytokines including TNF-α, IL-1, IL-6, and intercellular adhesion molecule-1 in lipopolysaccharide-challenged neuron (He et al., 2021). In another study, the expression of LncRNA NEAT1 was significantly increased in the temporal lobe tissue of epilepsy patients, and it could combine with miR-129-5p, resulting in an increased expression of key molecular in Notch signaling pathway and inflammatory cytokines such as IL-6 and TNF-α (Wan and Yang, 2020; see Table 1 for details).

**TABLE 1:** The pathways of LncRNAs involved in the pathogenesis of epilepsy.

LncRNAs	Expression changes	Functions	Patient samples or research models (Drugs)	References
H19		let-7b↓→STAT3, c-Myc↑→microglia and astrocyte activation↑	SD rats (KA)	Han et al., 2018a; Han et al., 2020
XIST		miR-29c-3p↓→NFAT5↑→neuronal apoptosis and neuroinflammation↑	SD rats (PTZ)/CTX-TNA (LPS)	Zhang et al., 2021
ILF3-AS1	↑	miR-212↓→MMP2, MMP3, MMP9, MMP14↑→neuroinflammation↑	Hippocampal tissue and serum from EPP/Astrocytes	Wan and Yang, 2020
NEAT1		miR-129-5p↓→Notch↑→neuroinflammation↑	Temporal lobe tissue from EPPs/CTX-TNA (IL-1β)	Wan and Yang, 2020
PVT1		BDNF↓/axin, cyclin D1↑→astrocyte activation↑	SD rats(LiCl/PILO)	Zhao et al., 2019
CASC2	↓	PTEN↓/p-P38, ADK, ENT1↑→astrocyte activation↑	SD rats (PTZ)/Primary rat astrocytes (PTZ)	Zhu et al., 2020
UCA1		JAK, STAT, Nrf2-ARE, GLAST↑→astrocyte activation↑	SD rats (KA)	Wang et al., 2020
		miR-203↑→MEF2C↓/NF-κB↑→neuroinflammation↑	SD rats (LiCl/PILO)/CTX-TNA (IL-1β)	Yu et al., 2020
GAS5		miR-219→CaMKIIγ/NMDAR→neuroinflammation↑	C57BL/6 mice (ATR/PILO)/SH-SY5Y (-Mg^2+^)	Zhao et al., 2022
TUG1		miR-199a-3p↓→neuronal apoptosis↑	Serum from EPPs/PHNs (-Mg^2+^)	Li et al., 2021
ZNF883		miR-181b↓→RASSF1A/MOAP1↑→neuronal apoptosis↑	PHNs (-Mg^2+^)	Gong et al., 2020
		miR-138-5p↓→USP47, NLRP3↑→neuronal apoptosis and inflammation↑	SD rats (ATR/PILO)/PHNs (-Mg^2+^)	Gong et al., 2022
SNHG1	↑	miR-154-5p↓→TLR5↑→neuronal apoptosis↑	Mice (LiCl/PILO)/SH-SY5Y (-Mg^2+^)	Zhao et al., 2020
H19		let-7b↓→Caspase3↑→neuronal apoptosis↑	SD rats (KA)	Han et al., 2018b; Sajadpoor et al., 2018
MALAT1		miR-101↓→c-Met↑/PI3K/akt↓→neuronal autophagy and apoptosis↑	SD rats (LiCl/PILO)	Wu and Yi, 2018
ZFAS1		miR-421↓→PI3K/Akt↓→neuronal autophagy and apoptosis↑	C57BL/6 mice (ATR/PILO)/PHNs (ATR/PILO)	Hu et al., 2020
		BCL2↓/BAX, Cas3, NF-κB↑→neuronal apoptosis and inflammation↑	Serum from EPPs/PHNs (-Mg^2+^)	He et al., 2021
FTX	↓	miR-21-5p↑→SOX7↓→neuronal apoptosis↑	SD rats (LiCl/PILO)/PHNs (-Mg^2+^)	Li et al., 2019
UCA1		miR-495↑→Nrf2↑→neuronal apoptosis↑	SD rats (LiCl/PILO)/PHNs (-Mg^2+^)	Geng et al., 2018
Nespas		miR-615–3p↓→Psmd11↓/PI3K/AKT/mTOR↑→neuronal apoptosis↑	C57BL/6 mice (ATR/PILO)/PHNs (ATR/PILO)	Feng et al., 2021
Peg13		miR-490-3p↑→Psmd11↓/β-catenin, cyclin-D1, c-Myc↑→neuronal damage and inflammation↑	C57BL/6 mice (ATR/PILO)	Feng et al., 2020
MEG3		PI3K/AKT/mTOR↓→neuronal apoptosis↑	SD rats (LiCl/PILO)/PHNs (-Mg^2+^)	Zhang et al., 2020

ATR, atropine; EPPs, epileptic patients; KA, kainic acid; LPS, lipopolysaccharide; PHNs, primary hippocampal neurons; PILO, pilocarpine; PTZ, pentylenetetrazol.

Nod-like receptor protein 3 (NLRP3) inflammasome-mediated inflammation has emerged as a contributor to epileptogenesis (Yue et al., 2020). Using mouse and cell epilepsy models, Zhang et al. (2022) found that NLRP3 inflammasome activation could enhance the expression of adenosine kinase and accelerate epilepsy *via* regulating CREB/REST/SP1 signaling pathway (Zhang et al., 2022). In line with these findings, Gong et al. reported that NLRP3 was increasingly expressed in epileptic neurons and rats, together with a downregulated expression of miR-138-5p and an upregulated expression of LncRNA ZNF883 and ubiquitin-specific peptidase 47 (USP47), and concurrent with aggravated inflammation and apoptosis (Gong et al., 2022). These findings suggested that inhibition of NLRP3 inflammasome might be a target for treatment of epilepsy. Furthermore, Gong et al. demonstrated a close relationship between LncRNA ZNF883 and NLPR3, *via* the regulation of USP47 and miR-138-5p in epilepsy models *in vivo* and *in vitro* (Gong et al., 2022). Moreover, four LncRNAs including Gm26917, Gm42418, Gm26767, and Neat1 were found to be enriched in NLRP3 inflammasomes (Zhang et al., 2019). Neat1, which was normally resided in the paraspeckles of macrophages, has been demonstrated to disassociate from these nuclear bodies and translocate to the cytoplasm under hypoxic conditions or stimulated with LPS, and modulate inflammasome activation via regulating caspase-1 activation, cytokine production, and pyroptotic cell death, or in a HIF-2α-dependent manner. Besides, LincRNA Cox2 has been reported to regulate NLRP3 inflammasome and autophagy mediated neuroinflammation through binding to NF-κB p65 and promoting its nuclear translocation and transcription (Xue et al., 2019).

### Long non-coding RNAs and neuronal apoptosis in epilepsy

Abnormal neuronal function and viability are the most important features of epilepsy. Increased neuronal apoptosis is strongly associated with epilepsy progression (Henshall and Simon, 2005). In a non-Mg^2+^ treated epileptic neuronal model and rat models induced by LiCl or pilocarpine, the expressions of LncRNA SNHG1 and LncRNA MALAT1 were significantly increased, while the expressions of LncRNA FTX and LncRNA MEG3 were significantly decreased (Wu and Yi, 2018; Li et al., 2019; Cai et al., 2020; Zhang et al., 2020).

Apart from promoting neuroinflammation, LncRNA ZFAS1 could also exacerbate epilepsy development via accelerating neuronal apoptosis. ZFAS1 expression in neurons was raised by pcDNA3.1- ZFAS1 and declined after silencing of ZFAS1 (He et al., 2021). Transfection of pcDNA- ZFAS1 could promote apoptosis (He et al., 2021), while knockdown of ZFAS1 could inhibit the apoptosis and autophagy of hippocampal neurons by activating the PI3K/AKT pathway via up-regulating miR-421 in epilepsy (Hu et al., 2020). LncRNA TUG1 could also regulate the cell activity and apoptosis of hippocampal neuron via sponging miR-199a-3p (Li et al., 2021). Besides the regulation effect of LncRNA 17A in autophagy and apoptosis, there were approximately 100 dysregulated LncRNA transcripts found in amyloid β peptide treated SH-SY5Y cells (Wang et al., 2019). LncRNA UCA1 could suppress pilocarpine-induced epilepsy by inhibiting apoptosis of hippocampal neurons via miR-495/Nrf2-ARE pathway (Geng et al., 2018). Furthermore, LncRNA H19 was highly expressed in the latent period of epilepsy, contributing to the apoptosis process of hippocampal neurons by targeting let-7b (Han et al., 2018b). Moreover, results of clinical observation indicated that the rs6478974 TT genotype could inhibit the susceptibility to epilepsy by reducing the levels of transforming growth factor beta receptor 1 (TGFBR1) mRNA, which is a target of let-7b (Zheng et al., 2021). In addition, the altered LncRNAs could involve with neuronal apoptosis by participating in the regulation of downstream oxidative stress and the expression of apoptosis-related proteins such as Nrf2, BCL2, and Caspase3 (Feng et al., 2020, 2021; Gong et al., 2020; Zhao et al., 2020; see Table 1 for details).

### Long non-coding RNAs and γ-aminobutyric acid/glutamate balance in epilepsy

Accumulating evidences from postmortem studies, brain imaging, and animal models demonstrate dysfunction of inhibitory GABAergic interneuron and excitatory Glu neurons contribute to epileptogenesis (De Deyn et al., 1990; Treiman, 2001; Pfisterer et al., 2020). The main clinical anticonvulsants, such as lacosamide, levetiracetam, phenobarbital, phenytoin, and valproate, are mainly targeting at improving the GABAergic activity or decreasing Glu-ergic activity (De Deyn et al., 1990). More importantly, vigabatrin, a second-generation antiepileptic drug with a remarkable effect against infantile spasms and focal seizures, is a structural analog of GABA that irreversibly inhibits GABA-transaminase (GABA-T) and increase brain levels of GABA (O’Callaghan et al., 2017; Knupp et al., 2022).

Highly expressed in the brain, LncRNAs are involved in important neurobiological process, including neurotransmitter synthesis and transmission, neurogenesis, and neural plasticity (Bond et al., 2009). It has been reported that the LncRNAs that associated with PICK1, GADL1, and PMD6 genes are abundant in the pathways involved with the ionotropic Glu receptor and GABA synthesis (Muniz et al., 2022). LncRNA Evf2 has been reported to play a crucial role in regulation of homeodomain transcription factors and the formation of GABA-dependent neuronal circuitry in the developing mouse forebrain, and LncRNA MALAT1 can modulate synaptic plasticity and neuronal regeneration via promoting the density of dendritic spines (Wu et al., 2013). Apart from abrogating the activation of astrocyte, over-expression of LncRNA UCA1 could suppresses the expression of astrocyte glutamate aspartate transporter (GLAST) via JAK/STAT signaling pathway, result in a reduced frequency of the of epilepsy seizures and a promotion of learning and memory in temporal lobe epilepsy rats (Wang et al., 2020). Besides, the regulation effect of LncRNA GAS5 in epilepsy was also involved in the regulation of Calmodulin-dependent protein kinase II (CaMKII)γ/*N*-methyl-D-aspartate receptor (NMDAR) pathway (Zhao et al., 2022). Moreover, non-coding RNA-dependent balanced gene regulation in embryonic brain has been reported to be critical for proper formation of GABA-dependent neuronal circuitry in adult brain (Bond et al., 2009). Furthermore, studies targeting at neuroblastoma have found that LINC00622 could promote GABBR1 expression via mediating the activity of transcriptional factor androgen receptor (Guo et al., 2022). Additionally, a dysfunction of LncRNAs/miRNAs/GABA regulatory axis has also been reported in depression animal model (Xue et al., 2022), and Glu decarboxylase-67 (Gad-67) and vesicular GABA transporter (VGAT) have been demonstrated to be directly regulated by miRNA-144-3p, miRNA-15b-5p, and miR-879-5p. Although LncRNA-mediated sponge regulatory network underlying GABA deficit in epilepsy remains unclear, a bidirectional relationship of epilepsy and depression or dementia has been demonstrated, involving both biological and psychosocial factors (De Deyn et al., 1990; Lothe et al., 2008; Sen and Romoli, 2021), sharing abnormalities in lots of neurotransmitters including Glu, with an antiepileptic activity of NMDA receptor antagonists (Lothe et al., 2008). Moreover, Valproic acid (VPA), a common anticonvulsant, has been reported to display an antitumor effect through regulating non-coding RNAs controlling gene expression *via* H19→EZH2→p21/PTEN pathway (Sajadpoor et al., 2018). Furthermore, it has been demonstrated that loss of LncRNA maternally expressed gene 3 (MEG3) could alleviate the impairment of learning and memory abilities of autism spectrum disorder rats induced by VPA, *via* promoting neuronal viability but inhibiting apoptosis (Liu X. et al., 2022). Although not based on epilepsy models, these findings indicated a close linkage between changes of LncRNAs and the GABA/Glu balance in epilepsy and the effect of anticonvulsants.

## Conclusion and outlook

Although a lot of energy and funds were invested in the research and development of antiepileptic drugs every year, the effective treatment for epilepsy are still unsatisfactory. Given the close linkage with neuroinflammation, apoptosis, and balance of transmitters especially GABA and Glu, LncRNAs might be taken as potential therapeutic targets for treatment of epilepsy. Though numerous LncRNAs have been identified, the mechanistic and functional roles for most LncRNAs are still unknown. Moreover, there is no reports about the dynamic changes of LncRNAs and their function in the progress of epilepsy, especially in different types of attacks. Thus, detailed investigation should be carried out not only about the changes of the abundance and diversity of LncRNAs in epilepsy, but the exact neurobiological mechanism as well, especially the mechanism about how they regulate the activity of transcription factors, through which how their regulations affect the downstream target genes expression.

## Author contributions

F-HC and J-FG proposed the conception of this work and supervised. SL and J-FG wrote the draft of the manuscript. SL, J-FG, and F-HC revised the manuscript. SL and MF searched literatures and analyzed the data. M-DM helped modifying the figure and table. All authors have read and approved the final manuscript.
